# Kinetic study of Pt nanocrystal deposition on Ag nanowires with clean surfaces via galvanic replacement

**DOI:** 10.1186/1556-276X-7-245

**Published:** 2012-05-06

**Authors:** Yu-Lin Shen, Shih-Yun Chen, Jenn-Ming Song, In-Gann Chen

**Affiliations:** 1 Graduate Institute of Applied Science and Technology, National Taiwan University of Science and Technology, Taipei, 106, Taiwan; 2 Department of Materials Science and Engineering, National Taiwan University of Science and Technology, Taipei, 106, Taiwan; 3 Department of Materials Science and Engineering, National Chung Hsing University, Taichung, 402, Taiwan; 4 Department of Materials Science and Engineering, National Cheng Kung University, Tainan, 701, Taiwan

## Abstract

Without using any templates or surfactants, this study develops a high-yield process to prepare vertical Ag-Pt core-shell nanowires (NWs) by thermally assisted photoreduction of Ag NWs and successive galvanic replacement between Ag and Pt ions. The clean surface of Ag nanowires allows Pt ions to reduce and deposit on it and forms a compact sheath comprising Pt nanocrystals. The core-shell structural feature of the NWs thus produced has been demonstrated via transmission electron microscopy observation and Auger electron spectroscopy elemental analysis. Kinetic analysis suggests that the deposition of Pt is an interface-controlled reaction and is dominated by the oxidative dissolution of Ag atoms. The boundaries in between Pt nanocrystals may act as microchannels for the transport of Ag ions during galvanic replacement reactions.

## Background

One-dimensional nanostructures have attracted much interest because of their fascinating properties and extensive use in electronics, sensing, catalysis, and electrochemical applications [1-4]. For example, changing the morphology of Pt nanostructure from nanoparticle to nanowire (NW) has been regarded as an important strategy to improve the performance of Pt-based catalysts, which have been widely used as the anode of direct methanol fuel cells for catalyzing the dehydrogenation of methanol [5-7]. By doing so, an enhancement in electrocatalytic activity can be obtained due to the large side surface, which is able to provide additional catalytic active facets. A great deal of effort has been devoted to the synthesis of Pt nanowires; however, it still remains a huge challenge to synthesize long and oriented single-crystalline Pt NWs [8-11]. Using template- and surfactant-free processes, the Pt NWs produced are extremely fine (mostly less than 10 nm in diameter) but exhibit a limit in length of about 200 nm.

Core-shell structure is an effective way to enhance the efficiency of precious metal electrocatalysts. In combination with the nanostructured core of less expensive or non-precious metals and the shell of Pt, high catalytic activity of core-shell bi-metallic electrocatalysts can be achieved. Previous studies have coated Pt on Pd, Au, Cu, and Ag nanowires, respectively, using PVD (magnetron sputtering) or galvanic deposition [12-15]. Excellent catalytic activity of those bi-metal NWs has also been demonstrated. Among these cases, the reports related to the synthesis of Ag-Pt core-shell nanowires are rare to our best knowledge. Zamborini et al. converted the surfaces of CTAB-protected Ag NWs into Pt by galvanic exchange reaction as shown below; however, the yield of nanowires thus produced was quite limited [13].

(1) $$2{\text{Ag}}^{\text{o}}{}_{\left(\text{s}\right)}+{\text{ PtCl}}_{4}{{}^{2-}}_{\left(\text{aq}\right)}\to {\text{Pt}}^{\text{o}}{}_{\left(\text{s}\right)}+2{\text{AgCl}}_{\left(\text{s or aq}\right)}+{\text{ 2Cl}}^{-},{\text{ E}}_{\text{o}}{}_{\text{cell}}=0.508\;\text{V}$$

A recently developed process, thermally assisted photoreduction, [16,17] has been successfully applied to prepare Ag nanowires with the length up to 10 μm on TiO_2_-coated substrates in large quantities without using templates and surfactants. The substrate could be rigid like Si wafers or flexible like carbon cloths. Ag nanowires thus produced are single-crystalline with a preferred <110> growth direction. The approach of this work is to use these clean-surface Ag NWs as sacrificial templates for the galvanic exchange with Pt ions to synthesize ultra-long Ag-Pt core-shell nanowires in large yield. In addition to the optical properties and microstructural characteristics, the mechanisms and reaction kinetics are also discussed.

## Methods

### Synthesis of Ag NWs on TiO_2_

**Figure 1:**
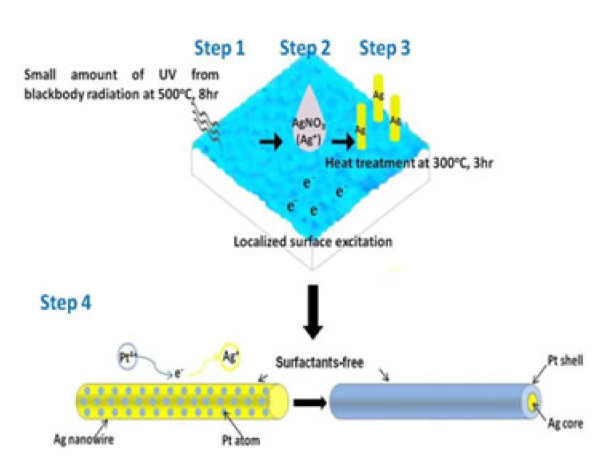
**Schematic illustration of the replacement process between Ag nanowires and Pt**^**4+**^**ions.**

The whole route for the synthesis of Ag-Pt core-shell NWs is illustrated in Figure 1. Gel-coating TiO_2_ films were synthesized on Si wafers and annealed at 500°C for 8 h in an oxygen atmosphere to obtain well-crystallized anatase TiO_2_ (step 1 in Figure 1). Fifteen microliters of 0.05 M aqueous AgNO_3_ solution was dropped on the TiO_2_-coated substrates (step 2). Afterward, the samples were isothermally heated at 300°C for 3 h in air by an infrared furnace, followed by furnace cooling to ambient temperature (namely, the post thermal treatment; step 3 in Figure 1).

### Galvanic exchange between Ag and Pt salts

Ag NWs thus prepared were removed from the TiO_2_ substrate by ultrasonic oscillation and then immersed in 0.05 M aqueous Na_2_Pt(OH)_6_ solution for the exchange of Pt (step 4 in Figure 1). Instead of commonly used precursor, H_2_PtCl_6_, Na_2_Pt(OH)_6_ was adapted in this study because Pt ions can be reduced more easily mainly due to the smaller electronegativity of the OH group (3.02) [18,19] compared with Cl (3.16). The solution was then isothermally heated to reflux at 100°C, 170°C, and 200°C. After a certain reaction period, the samples were rinsed with deionized water and dried under N_2_ before analysis.

### Characterizations of core-shell NWs

The structure and phase of the NWs were characterized by transmission electron microscopy (TEM) (G_2_, Philips Tecnai, Amsterdam, The Netherlands) with an accelerating voltage of 200 kV and also a grazing incidence X-ray diffraction meter (D/MAX2500, Rigaku Corporation, Tokyo, Japan) (incidence angle of 0.5°) with graphite monochromatic CuKα radiation (*λ* = 0.15418 nm) at a scanning rate of 2° per min from 30° to 80°. The morphology and size distribution were obtained by scanning electron microscopy (SEM) (JSM-6700, JEOL Ltd., Akishima, Tokyo, Japan) with an accelerating voltage of 20 kV. The measurements of the UV-visible (vis) absorption spectra were carried out at room temperature using a Hitachi-4001 spectrophotometer (Hitachi High-Tech, Minato-ku, Tokyo, Japan). Auger electron spectroscopy (AES) (MICROLAB 350, Thermo VG Scientific, West Sussex, England) equipped with an Ar ion gun was used to investigate the elemental distribution of the cross-sectioned nanowires.

## Results and discussion

### Morphology and structure of Ag NWs

**Figure 2:**
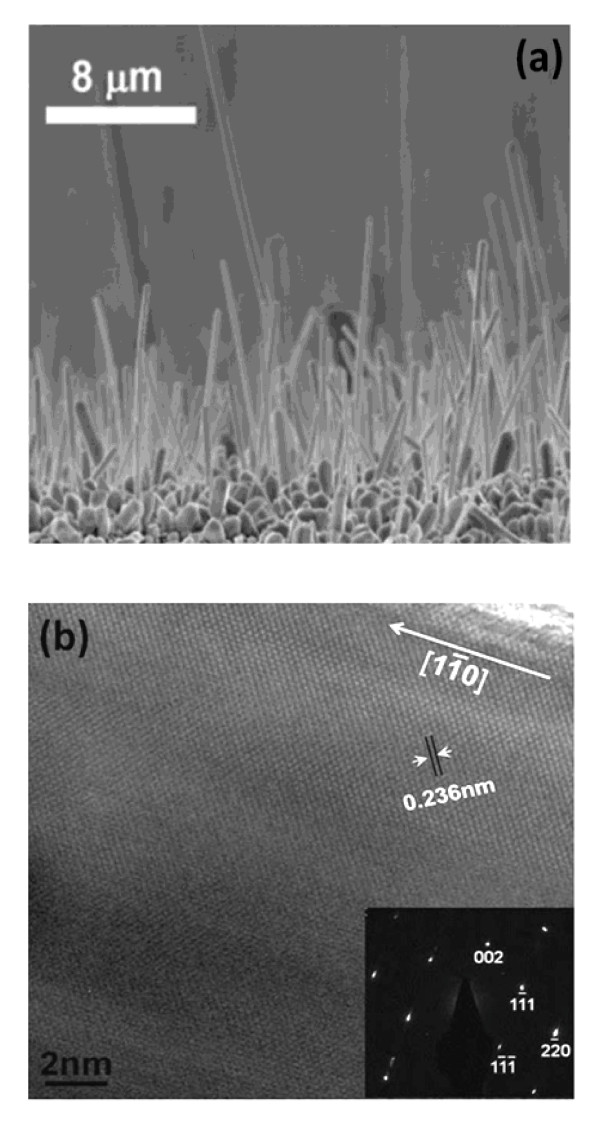
**Vertically grown Ag nanowires.** (**a**) SEM images, (**b**) HRTEM lattice image, and the corresponding electron diffraction patterns by fast Fourier transform.

The SEM image in Figure 2a shows the Ag NWs synthesized by heating aqueous AgNO_3_ droplets on the TiO_2_ substrate at 300°C for 3 h. It reveals that the Ag NWs were well-aligned and had an average diameter and length of about 100 nm and 10 μm, respectively. The yield of NWs (wire density) was up to 70 NWs/100 μm^2^. The high resolution TEM (HRTEM) image and selected-area electron diffraction (SAED) pattern shown in Figure 2b confirm that the NW was single crystalline Ag with $1\bar{1}0$ growth direction. The growth texture can be ascribed to the difference in the surface energy. The Ag {110} surfaces possess the highest surface energy of 770 mJ/m^2^, while those for {100} and {111} surfaces are 705 and 620 mJ/m^2^, respectively [20]. To lower the total free energy, atoms tend to accumulate along the normal direction of the {110} plane.

### Optical and structural characteristics of Ag-Pt core-shell NWs

**Figure 3:**
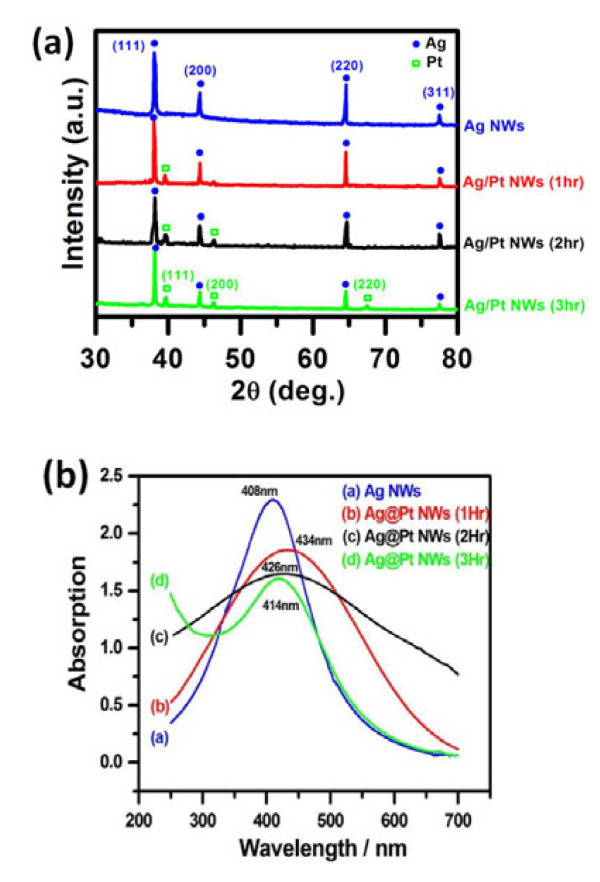
**XRD patterns and UV–vis spectra of A-g-Pt core-shell nanowires.** (**a**) XRD patterns and (**b**) UV–vis spectra of Ag-Pt core-shell nanowires with different reaction times at 170°C.

X-ray diffraction (XRD) patterns of the Ag NWs subjected to reaction with the Pt salt solution at 170°C as a function of time are shown in Figure 3a. Pt (111) peak emerged after being reacted for 1 h. After that, more Pt signals can be identified in the extended reaction because of the increasing amount of Pt. Pt (200) peak was observed after a 2-hr reaction, and that of Pt (220) could be detected after 3 h. Figure 3b illustrates the corresponding absorption spectra. As for the unreacted NWs (curve a), the observed plasma resonance band with a wave length of about 408 nm is due to the absorption of one-dimensional Ag nanostructures [21,22]. The decreased intensity as well as a small red shift of this absorption peak occurred after galvanic exchange. Subjected to a 3-h exchange with Pt salts, one additional peak appeared at the extremely low wavelength side, which is believed to be due to the deposition of Pt [23,24].

**Figure 4:**
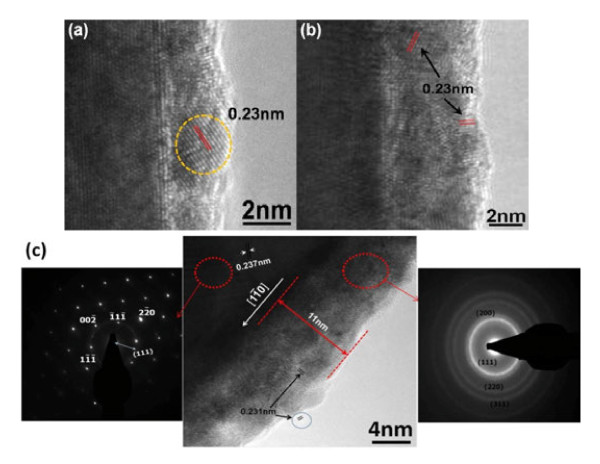
**TEM images showing Pt nanocrystals deposited on surface of Ag wires with different reaction times.** Different reaction times at 170°C, (**a**) 1 h, (**b**) 2 h, and (**c**) 3 h, as well as the SAED patterns of Ag core and Pt shell.

To further verify the precipitation of Pt on the surface of Ag NWs, TEM and AES were applied for microstructural examination. HRTEM images taken from the near-edge regions of the NWs after different periods of reaction time (Figure 4) indicate that the deposits were compact and attached firmly to the Ag surface. Their layer thickness increased with a prolonged reaction time. It is noteworthy that the right diffraction pattern in Figure 4c, as well as the measured lattice spacing of 0.23 nm corresponding to the distance between the {111} planes of Pt, suggests that the deposits obtained from galvanic exchange were composed of numerous Pt nanocrystals. Again, the left diffraction pattern verifies that what the Pt nanocrystals covered densely is a single crystalline Ag NW. The above results may somewhat reveal that without surfactants and grain boundaries, clean surface of single crystalline Ag NWs is favorable for the precipitation of Pt.

**Figure 5:**
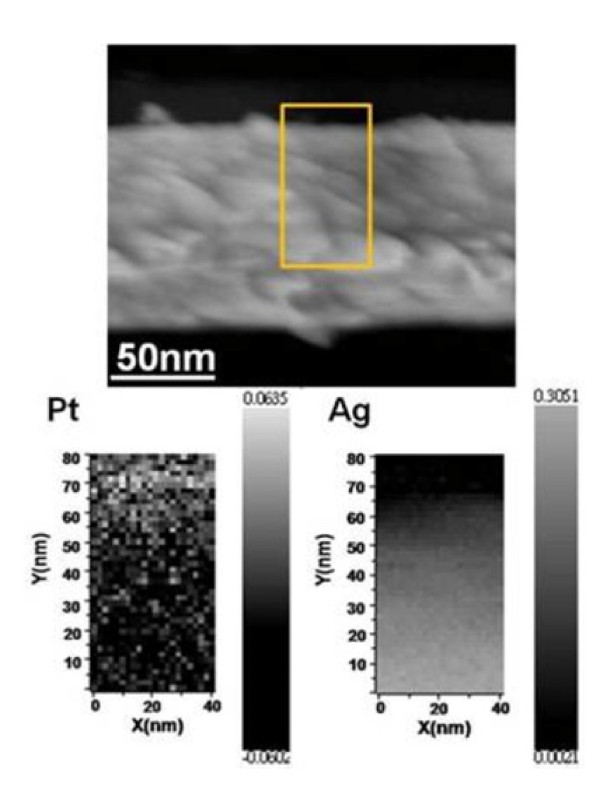
An ion-etched Ag-Pt core-shell nanowire (300 s) and Auger electron elemental mapping of the circled area.

By means of ion etching, a core-shell nanowire was cross-sectioned (as shown in Figure 5), and the elemental analysis of the circled area on the sectioned surface was performed using AES. The Pt mapping indicates that the near-edge region was enriched in Pt, while the Ag signal could only be detected in the inner region. The very weak Pt signal in the inner region can be considered as the contamination by ion etching. The elemental mapping data further certify the core-shell structural feature of the galvanic-reacted NWs.

### Reaction kinetics and mechanisms

**Figure 6:**
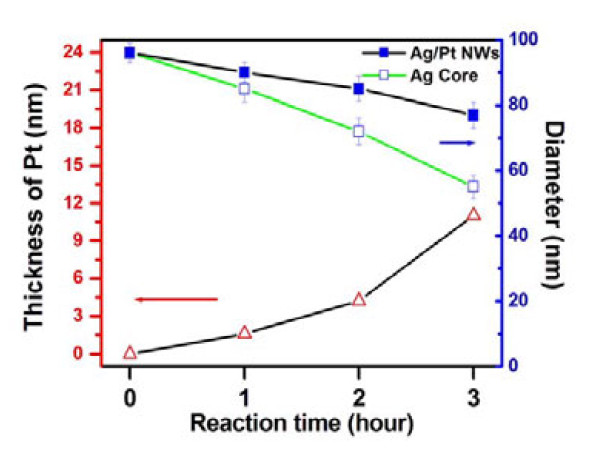
Variation in thickness of Pt shells and diameters of Ag cores and core-shell wires at 170°C.

Kinetic data given in Figure 6 depict the changes in the thickness of the Pt shell and also the diameter of NWs at 170°C. The diameter of the Ag core was estimated by the difference between the diameter of NWs and the Pt thickness on both sides of the core-shell NWs. It can be found that with a prolonged reaction time, Pt became thickened along with the shrinkage in the Ag core. Interestingly, the diameter of the whole core-shell NWs also shrunk with the reaction time.

**Figure 7:**
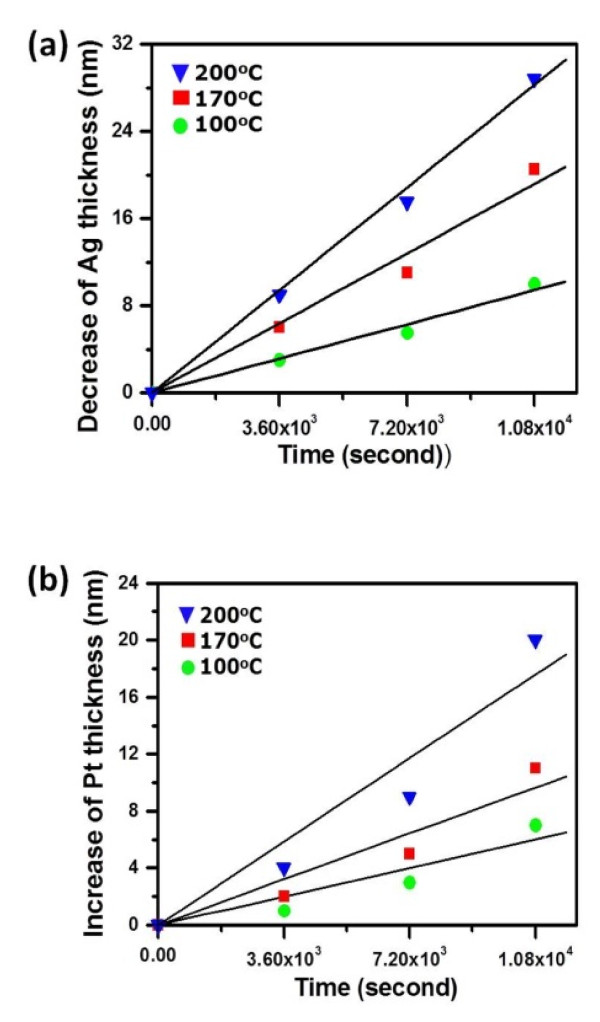
**Quantitative and kinetic analyses at different reaction temperatures.** (**a**) The decrease of Ag and (**b**) the gain of Pt.

The decrease in the Ag core diameter as well as the increase in thickness of Pt deposits as a function of reaction time and temperature was illustrated in Figure 7. According to following equations, the kinetics of galvanic reactions can be analyzed [25]:

(3) $$h=k_{0}t^{n}$$

(4) $$k_{0}=k_{1}e^{-Q/RT}\text{,}$$

where *h* is the decrease in Ag wire diameter or increase in Pt layer thickness; t is the reaction time; *Q* is the activation energy; *R* is the gas constant; *T* is the absolute temperature; and *k*_0_ and *k*_1_ are constants. Regression analysis reveals that both of them followed a linear relation with the reaction time (i.e., *n* = 1). This implies that the growth of Pt and the consumption of Ag are both interface controlled.

**Figure 8:**
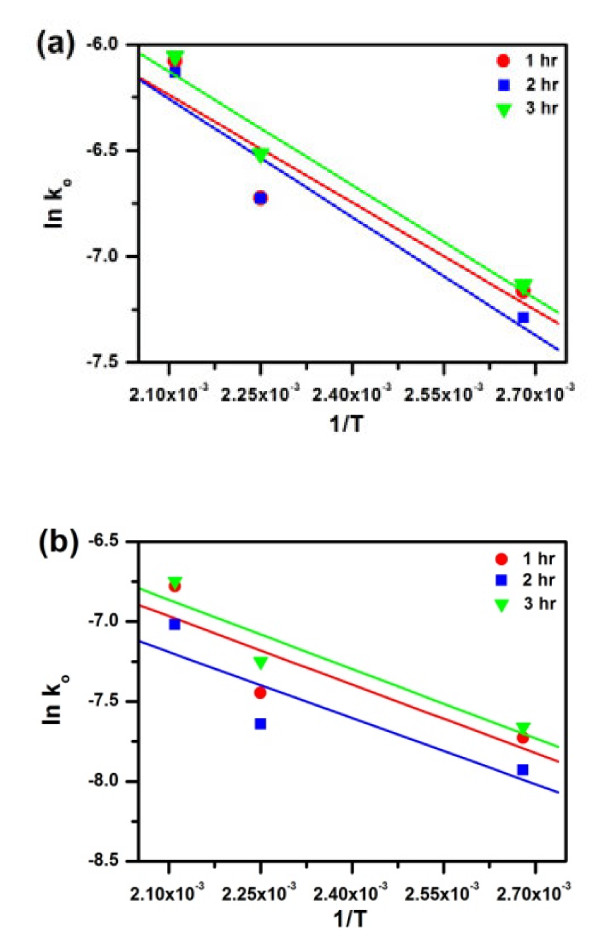
**Arrhenius plot of the rate constant as a function of the reciprocal of reaction temperature.** (**a**) The decrease of Ag and (**b**) the gain of Pt.

The values of ln *k*_0_ are plotted against the inverse temperature (1/*T*) in Figure 8 to calculate the activation energy. From the slopes in Figure 8, the activation energies estimated are listed in Table 1. As tabulated, activation energy for the reduction of Pt is about 11.7 kJ/mol (0.123 eV/atom), while that for the oxidation decomposition of Ag is about 15 kJ/mol (0.157 eV/atom). It is obvious that the dissociation of Ag into electrons and ions encounters greater energy barrier. The fact that the activation energy values derived from different reaction times (Table 1) are quite similar may again prove that galvanic exchange of Ag by Pt is said to be interface controlled rather than diffusion controlled.

**Table 1:** Activation energies of the reactions in galvanic replacement

	**Decrease in Ag**		**Gain of Pt**	
	***Q*(kJ/mol)**	***Q*(eV/atom)**	***Q*(kJ/mol)**	***Q*(eV/atom)**
1 h	15.03	0.157	11.86	0.124
2 h	15.36	0.160	11.38	0.119
3 h	14.69	0.153	11.95	0.125

In the present study, the redox reaction along with those for the anodic dissolution and cathodic reduction are given as follows:

(5) $${4\text{Ag}}_{\left(\text{s}\right)}+\text{Pt}{\left(\text{OH}\right)}_{6}{{}^{2-}}_{\left(\text{aq}\right)}+6\;{\text{H}}^{+}\to {4\text{Ag}}^{+}{}_{\left(\text{aq}\right)}+{\text{Pt}}_{\left(\text{s}\right)}+{6\text{H}}_{2}{\text{O}}_{\left(\text{aq}\right)},{\text{ E}}_{\text{ocell}}=0.38\;\text{V}$$

Dissolution of Ag:

(6) $$4\text{Ag}\to {4\text{Ag}}^{+}+{4\text{e}}^{-}(-0.8\;\text{V vs SHE})$$

Reduction of Pt ions:

(7) $$\text{Pt}{\left(\text{OH}\right)}_{6}{}^{2-}+{4\text{e}}^{-}+6\;{\text{H}}^{+}\to \text{Pt}+{6\text{H}}_{2}\text{O }(1.18\;\text{V vs SHE})\text{,}$$

where SHE is the standard hydrogen electrode.

**Figure 9:**
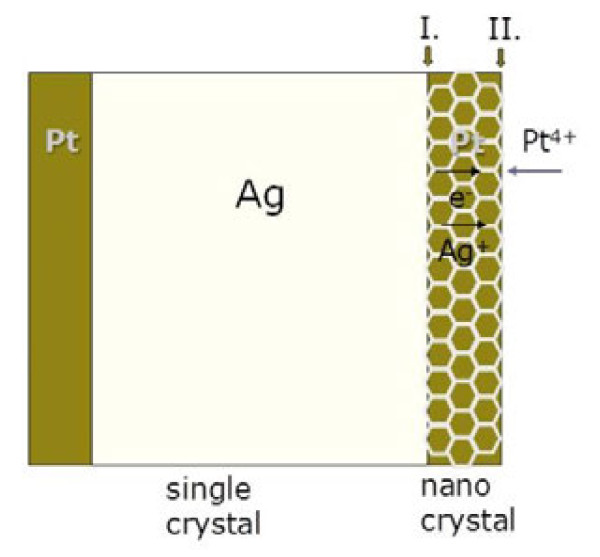
Schematic illustration of galvanic replacement of Ag by Pt ions to form a core-shell structure.

Figure 9 delineates the processes for the galvanic replacement in question. As aforementioned, Pt was deposited on the surface of Ag wires as a compact layer of nanocrystals. It can be suggested that there were two interfaces: the inner surface of the Pt deposit between Ag and reduced Pt (interface I in Figure 9) and the other one, the outer surface between reduced Pt and solution (interface II). Dissociation of Ag (Equation 5) can be considered to occur at interface I, while the reduction of Pt took place at interface II (Equation 6). There is no evidence so far showing the orientation relationship between single crystalline Ag and Pt nanocrystals. However, it can be inferred that boundaries in between Pt nanocrystals provided large quantities of microchannels for the transportation of Ag ions, and thus, Ag ions generated at interface I could transfer rapidly through the Pt deposit. On the other hand, the electrons could travel through highly conductive Pt deposits easily to support the reduction of Pt ions at interface II. All of these can explain why the galvanic deposition process is interface controlled instead of diffusion controlled. Again, according to the kinetic analysis above, the dissociation reaction of Ag at interface I dominated this deposition process.

## Conclusions

In this study, surfactant-free single crystalline Ag NWs prepared by thermally assisted photoreduction were used as sacrificial templates for the galvanic deposition of Pt. Through this simple route, large quantities of vertical Ag-Pt core-shell NWs could be prepared. Due to the great reduction potential, Pt ions could be reduced by the electrons generated from the oxidative dissolution of Ag. A compact shell comprising Pt nanocrystals can thus be formed on the surface of Ag NWs. TEM observation and AES elemental mapping both verify the core-shell structural feature of these NWs. According to the kinetic analysis and estimated activation energy, the galvanic exchange of Ag by Pt ions was an interface-controlled process, which was dominated by the dissociation of Ag atoms.

## Competing interests

The authors declare that they have no competing interests.

## Authors' contribution

Y-LS carried out the main part of synthetic and analytical works, participated in the sequence alignment, and drafted the manuscript. S-YC participated in the discussion of the growth mechanism. J-MS participated in the design of the study and in draft preparation and coordination. I-GC conceived the study and participated in its design. All authors read and approved the final manuscript.

## Authors' information

Y-LS is a Ph.D. student at the Graduate Institute of Applied Science and Technology, National Taiwan University of Science and Technology, Taipei, Taiwan. S-YC is an associate professor of the Department of Materials Science and Engineering, National Taiwan University of Science and Technology, Taipei, Taiwan. J-MS is an associate professor of the Department of Materials Science and Engineering, National Chung Hsing University, Taichung, Taiwan. I-GC is a distinguished professor at the Department of Materials Science and Engineering, National Cheng Kung University, Tainan, Taiwan.
